# The nature of the diffuse light near cities detected in nighttime satellite imagery

**DOI:** 10.1038/s41598-020-64673-2

**Published:** 2020-05-08

**Authors:** Alejandro Sanchez de Miguel, Christopher C. M. Kyba, Jaime Zamorano, Jesús Gallego, Kevin J. Gaston

**Affiliations:** 1 Environment and Sustainability Institute, University of Exeter, Penryn, Cornwall, TR10 9FE UK; 2 Department Física de la Tierra y Astrofísica and Instituto de Física de partículas y del Cosmos IPARCOS, Universidad Complutense de Madrid, Madrid, 28040 Spain; 3 Instituto de Astrofísica de Andalucía, Glorieta de la Astronomía, s/n,C.P, 18008 Granada, Spain; 4 GFZ German Research Centre for Geosciences, 14473 Potsdam, Germany; 5 Leibniz-Institute of Freshwater Ecology and Inland Fisheries, 12587 Berlin, Germany

**Keywords:** Ecological epidemiology, Environmental impact, Astronomical instrumentation, Atmospheric optics

## Abstract

Diffuse glow has been observed around brightly lit cities in nighttime satellite imagery since at least the first publication of large scale maps in the late 1990s. In the literature, this has often been assumed to be an error related to the sensor, and referred to as “blooming”, presumably in relation to the effect that can occur when using a CCD to photograph a bright light source. Here we show that the effect seen on the DMSP/OLS, SNPP/VIIRS-DNB and ISS is not only instrumental, but in fact represents a real detection of light scattered by the atmosphere. Data from the Universidad Complutense Madrid sky brightness survey are compared to nighttime imagery from multiple sensors with differing spatial resolutions, and found to be strongly correlated. These results suggest that it should be possible for a future space-based imaging radiometer to monitor changes in the diffuse artificial skyglow of cities.

## Introduction

**Figure 1:**
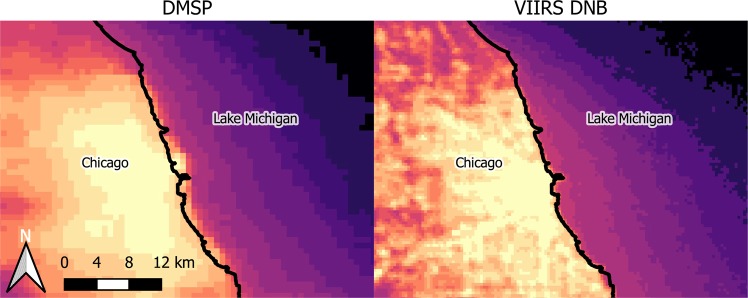
Images of the area near the city of Chicago, Illinois, using EOG’s radiance calibrated DMSP composite from 2010 (left) and the VIIRS-DNB composite from October 2012 (right). The black line marks the shore of the lake. Note that the glow extends well out onto Lake Michigan (right side), an area with little or no light emission. The color scales are both logarithmic, but are not identical (DMSP and VIIRS-DNB data cannot be directly compared). See also Figs. 10 and 12 in Levin 2017^59^.

A diffuse glow of light surrounding cities, such as that seen in Fig. 1, was noted by the Earth Observation Group (EOG) who were then at the National Oceanic and Atmospheric Administration from their earliest publications of nighttime light data sets (e.g^1,2^.). Due to the low spatial resolution of the Defence Meteorological Satellite Program Operational Linescan System (DMSP), it was not possible to determine the nature of these observations, and in particular whether they were instrumental artifacts or real observations of light. Since that time, many publications have referred to this glow as “blooming”, perhaps by analogy to the effect seen near bright light sources in early CCD imagery (e.g.^1,3^). Regardless of its cause, this glow presents a problem for some analyses (e.g. detection of urban areas^4,5^), and has therefore frequently been effectively treated as an instrumental error. Newer satellite data are no longer restricted by the saturation problems and low spatial resolution of DMSP data, but still contain the glow near cities (Fig. 1). This strongly suggests that the glow is not an instrumental effect, but rather real light being detected by the instruments.

Observers from the ground have long been aware of another type of glow near cities, the artificial sky brightness known as “skyglow”, a form of light pollution^6–8^. Skyglow is caused when light that is emitted upward (or reflected from the ground) scatters off molecules or aerosols in Earth’s atmosphere, and is re-directed towards the ground^9^. Systematic academic study of skyglow began in the 1970s^10–13^, and physical modeling of skyglow began in the 1980s^14,15^; we recently learned that the first experimental studies of skyglow were conducted by the US War Department during World War 2^16^. Could the diffuse glow around cities in satellite imagery be caused by the same effect?

**Figure 2:**
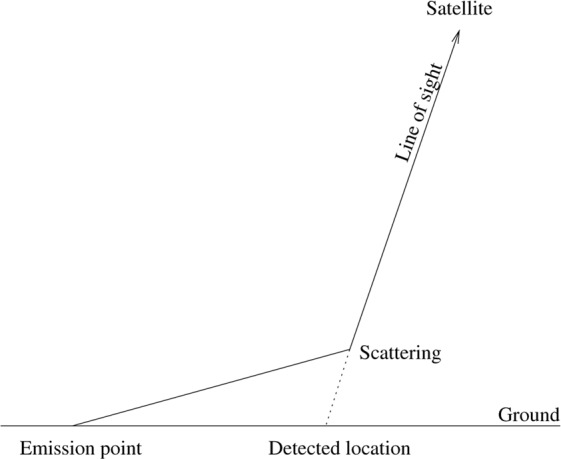
Schematic diagram showing how scattered light is falsely attributed to a location in nighttime satellite imagery. Light emitted from the emission point undergoes a scattering, changing its direction. The satellite radiometer viewing along the direction that the light was propagating attributes it to a false location.

Figure 2 demonstrates how scattered light is attributed to an incorrect location in observations of night lights from space. The scattered light appears to come from a position on the line of sight, rather than from its true emission location. This effect could therefore explain, for example, the detection of light seeming to emanate from Lake Michigan in regions near Chicago (Fig. 1). But how can we be sure that this is the cause, and if it is, what is the relationship between the skyglow observed from the ground and that observed from space?

Rayleigh scattering has both forward-backward and azimuthal symmetry, so the fraction of light scattered back towards Earth by molecules must be nearly equal to the amount scattered upwards (ignoring a minor difference caused by the curvature of the Earth). Aerosols primarily scatter light through small angles in the forward direction. For upward directed light in general, one would therefore expect a difference in their contribution to skyglow observed from space compared to the ground. However, the main contributor to skyglow observed from the ground is light that is emitted at near-horizontal angles^9,16,17^. For horizontally directed light, the azimuthal symmetry of Mie scattering implies that a similar amount of large angle scatter would occur in both the upward and downward directions. Taken together, these facts imply that if atmospheric scattering is the cause of the glow around cities, one must expect a fairly strong relationship between skyglow observed from the ground and the glow in satellite imagery.

Kyba *et al*. and Zamorano *et al*. have previously examined the relationship between ground based observations of skyglow and the DMSP data^18,19^ using Sky Quality Meters (SQM^20–22^). Much of the data used in Kyba *et al*. were from lit locations, and they dismissed the possibility that the DMSP values directly represented skyglow. Zamorano *et al*., on the other hand, took data primarily from locations with no installed lighting, and explicitly raised the possibility of this connection^18^. In this work, we use data taken in areas away from installed lighting to test the relationship between skyglow observed from the ground and from space. We used ground data from the Universidad Complutense Madrid sky brightness survey. In order to guarantee that these sky brightness measurements were not contaminated by spurious lighting, a manual inspection of all the data was performed. No data was considered that was taken closer than 20 m from a street light or inside a city, and all measurements brighter than 18 mag_SQM_/arcsec^2^ were rejected with the exception of handheld measurements taken from the top of buildings above the level of street lights. We used measurements taken from space by the DMSP, and data with higher resolution and sensitivity from the Suomi North Polar Partnership (SNPP) Visible Infrared Imaging Radiometer Suite (VIIRS) Day/Night Band (DNB) and astronaut photographs from the International Space Station (ISS)^23–26^. In the case of the satellite data, we only considered areas with sky brightness (SB) darker than 18.5 mag_SQM_/arcsec^2^.

## Results

**Figure 3:**
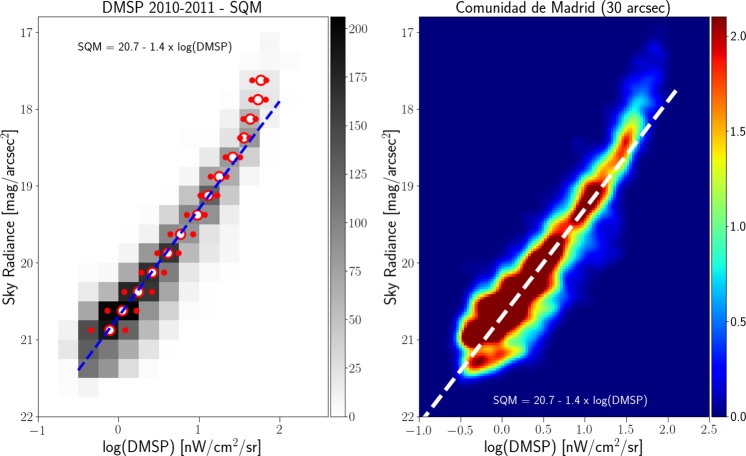
Correlation between the sky brightness at zenith (UCM sky brightness survey) and the DMSP year average saturation corrected 2011 radiance calibrated data for the region of Madrid. The ground-based data have been averaged on the same 30 arcsec grid as the DMSP data. The left hand plot is a 2D histogram of the radiance as measured from Earth and from space. The number of 30 arcsec cells for each bin is coded as gray shades. In the left plots, the green circle represents the dimmer Gaussian peak, and the red circles the brighter Gaussian peak. The dashed blue line is a linear fit using the darkest values and rejecting the bins brighter than 19 mag/arcsec^2^. The relationship is SB = (−1.40 ± 0.02)log_10_(DMSP) + 20.71 ± 0.01 with *R*^2^ = 1 for the median values with sky brightness higher than 19. The right hand panel shows the same result using a non-binned color coded density plot with a Gaussian filter. The dashed white line reproduces the fit of the left panel and has been added for comparison purposes.

**Figure 4:**
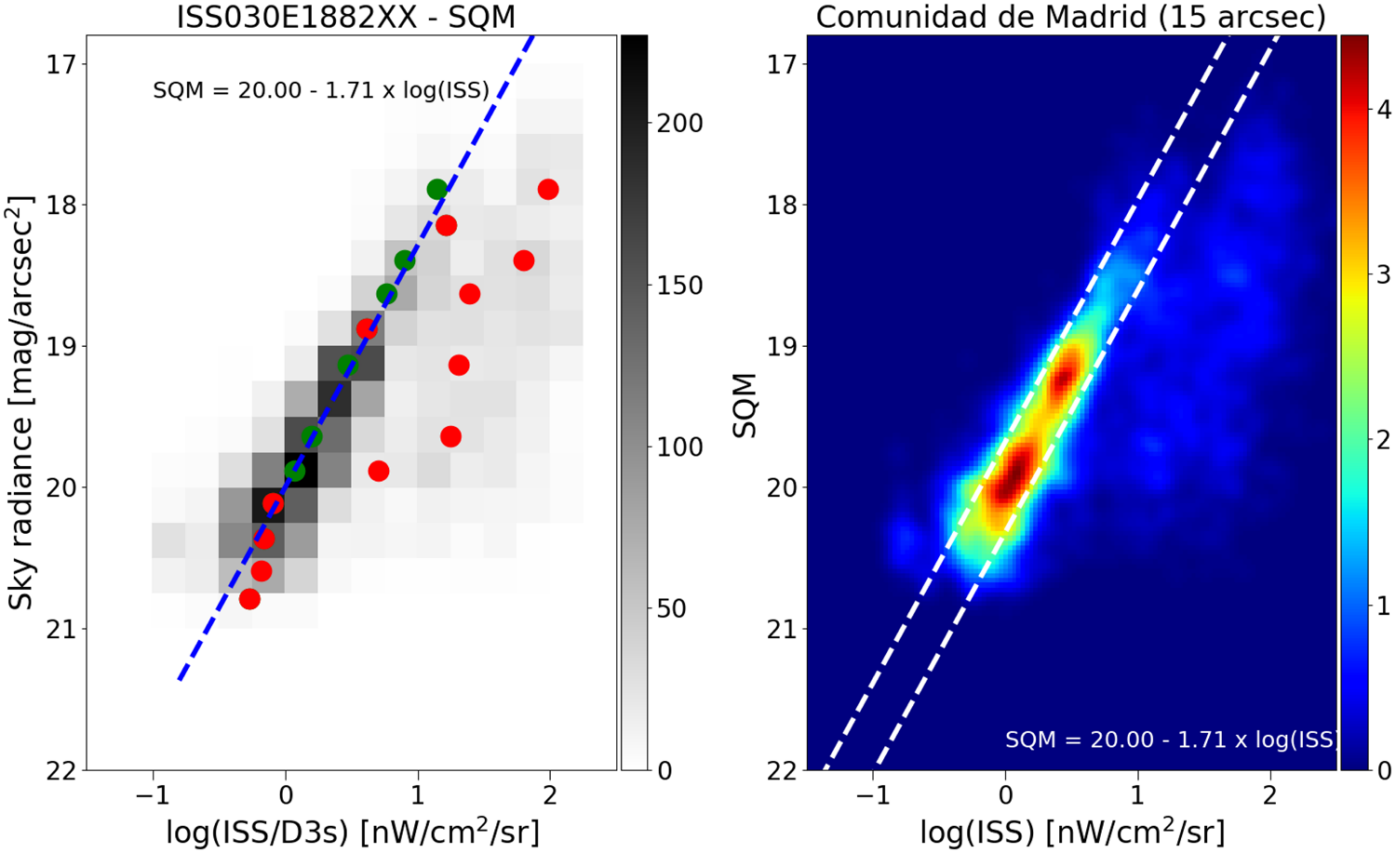
Correlation between the sky brightness at zenith (UCM sky brightness survey) and a composite high dynamic range image produced from astronaut photographs (ISS030E188208 and ISS030E188210) for the region of Madrid. The individual ground observations and the ISS image have been averaged on the same 15 arcsec grid. The left hand plot is a 2D histogram of the radiances as measured from Earth and from space. The number of 15 arcsec cells for each bin is coded as gray shades. The open dots and smaller red dots represent the median value for each sky radiance bin and the 25% and 75% percentiles respectively. The dashed blue line is a linear fit using the darkest values and rejecting the bins brighter than 18.8 mag/arcsec^2^. The relationship is SB = (−1.71 ± 0.1)log_10_(ISS) + 20.00 ± 0.05 with *R*^2^ = 0.98 for the median values with sky brightness higher than 18.5. The right hand panel shows the same result using a non-binned color coded density plot with a Gaussian filter. We have added two dashed white lines with the same slope as the blue line in the left panel for comparison purposes.

There is a strong correlation between the zenith night sky radiance data from the Universidad Complutense de Madrid (UCM) sky brightness survey (see Methods) and the space-based datasets with both low (Fig. 3) and high resolution (Fig. 4). Sky radiance is shown in mag_SQM_/arcsec^2^, an astronomical unit for which larger values represent darker skies (see Methods; Puschnig *et al*.^27,28^, and Sánchez de Miguel *et al*.^29^). Note that the radiance observed by DMSP and photographs from the ISS cannot be directly compared, because of the different spectral sensitivity of the instruments^30^.

**Figure 5:**
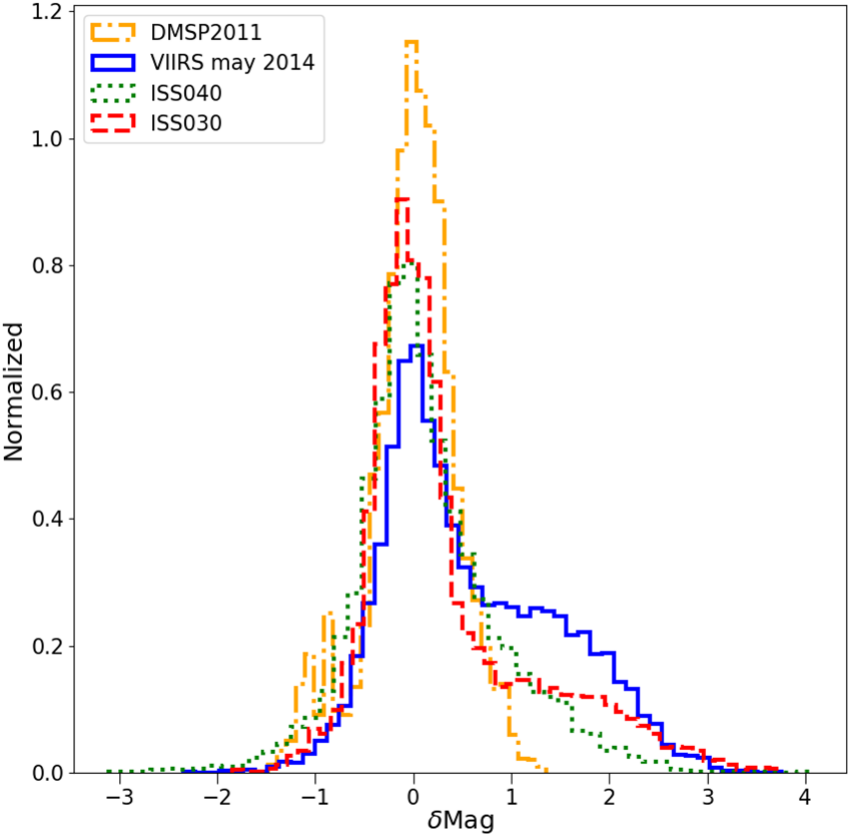
Histogram of the difference in observed skyglow radiance from the ground compared to the prediction from the space based observation. Positive values indicate a darker sky than predicted. The imagery is listed in order of decreasing resolution. The histograms have been normalized (the area under the histogram will sum to 1).

For each space-based observation, we performed a least squares regression to predict the (logarithmic) Sky Quality Meter (SQM) derived radiance (mag_SQM_/arcsec^2^) based on the logarithm of the radiance observed by satellite (in nW/cm^2^sr). With this fit, it is then possible to compare the individual observations to the prediction to examine the dispersion of the data. The fit residuals (observed minus predicted radiance in mag_SQM_/arcsec^2^) are shown for each of the four space-based observations in Fig. 5. The satellite observations have an intrinsic resolution of 5 km (DMSP), 750 m (DNB), 240 m (ISS040-E-08258-62), and 54 m (ISS030-E-188208-10).

**Figure 6:**
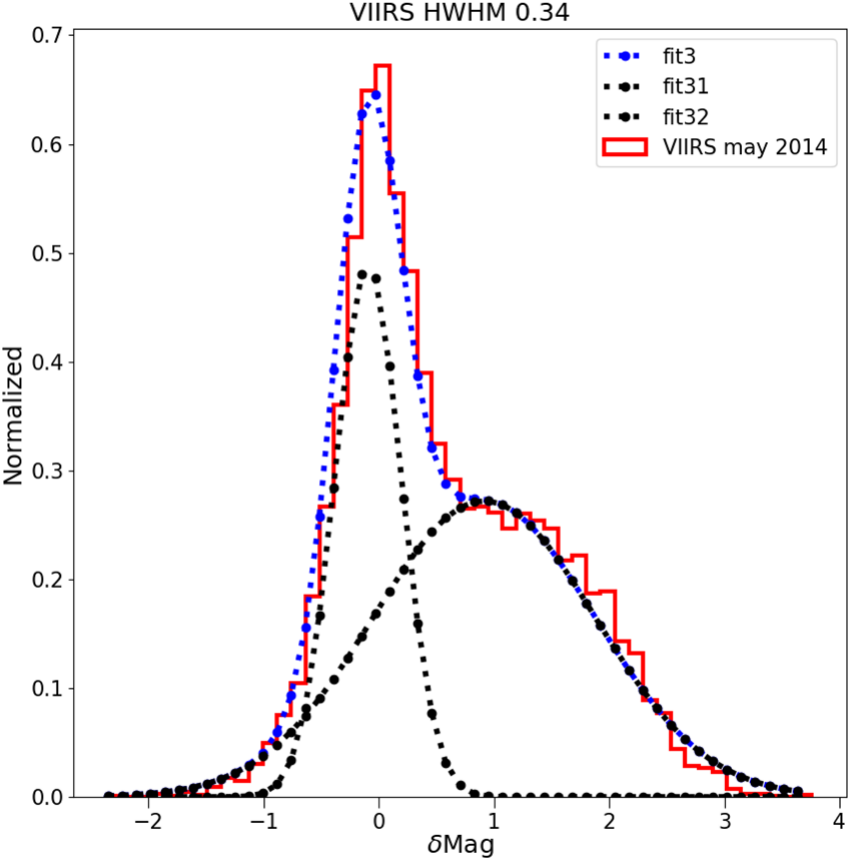
Example of double Gaussian fit to the residual of sky brightness for the data from VIIRS-DNB. The narrow peak at left represents observations in areas with no installed lighting, the peak at right is areas in which the areas contain a mix of both sky brightness and light emissions from the ground.

It is clear that for the high resolution imagery data (see Fig. 4), there is a set of data on the high side, meaning that the sky is darker than we would expect based on the space-based image. These data are fairly well described by a double Gaussian fit (e.g. Fig. 6). The half width at half maximum of the main component (taller left hand peak) of the double Gaussian fit to the residual histograms is about ~0.35 mag_SQM_/arcsec^2^ for each of the space-based datasets. In terms of radiance, this corresponds to a typical difference of roughly ±35%. We believe that the second Gaussian distribution is caused by pixels which contain a mixture of both upward scattered skyglow and direct light emissions from the ground, whereas the first Gaussian represents pixels which contain only upward scattered skyglow.

**Figure 7:**
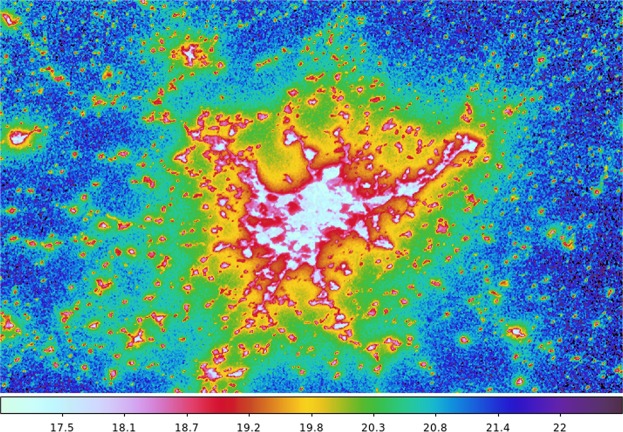
Sky brightness map of the central area of Spain, centered on the city of Madrid, based on the correlation between the light scattered up and the light scattered down. This model is using the HDR ISS data. VIIRS interactive version, at URL: https://pmisson.users.earthengine.app/view/trends. Scale in *mag*/*arcsec*^2^.

These results suggest that it should be possible to create maps of regional sky brightness, or even global sky brightness maps based on real VIIRS measurements. We have done this for the Madrid area (Fig. 7). We have also started work on a project to produce a world map using Google Earth Engine^31^. A preliminary version of this map can be examined at URL: https://pmisson.users.earthengine.app/view/trends. We intend to improve upon this original effort in the future, once new and better calibrations of VIIRS and sky brightness databases are made publicly accessible.

### Relationship between sky brightness and diffuse light

Correlations between top of atmosphere radiance and sky brightness were observed for each satellite dataset, despite their very different spatial resolutions. Furthermore, the correlations in regions with no direct illumination that correspond to the main Gaussian component have similar dispersion. This is consistent with the hypothesis that the diffuse glow observed in the images is due to real light. It is also consistent with the hypothesis that at distances of at least several hundred meters from light sources, the upward directed artificial glow of the night sky is directly proportional to the downward directed glow. The fit parameters for the different satellite sensors are not identical. Note, however, that the acquisitions are fundamentally different: DMSP is based on an annual composite, VIIRS on a monthly composite, and the ISS data are taken instantaneously (from two nights). The atmospheric conditions (e.g. aerosol properties) therefore differ between the images. Finally, the four sensors (SQM, DMSP, VIIRS and ISS) have different spectral responses.

### Comparison with the world sky brightness atlas

The World Atlas of Artificial Night Sky Brightness first version was released in 2001^32^ and a new version was released in 2016^33^. The more recent version was calibrated with 20,865 observations from all over the world thanks to the citizen science program Globe at Night (GaN) and 10 other datasets including considerable observations from Madrid, Berlin, and Catalonia. The Madrid data and much of the Berlin data were collected with the same instruments as the UCM Sky brightness Survey, with a similar half width at half maximum to that reported in this paper. The World Atlas used a radiative transfer model to predict the artificial light at zenith, assuming constant atmospheric conditions and correcting for the elevation of the location. Although there are maps generated by other sky brightness models^34–37^, these either do not have associated data validation or their validation has been done locally. Currently, Falchi *et al*.^33^ is the best and most extensively validated artificial sky brightness map available.

**Figure 8:**
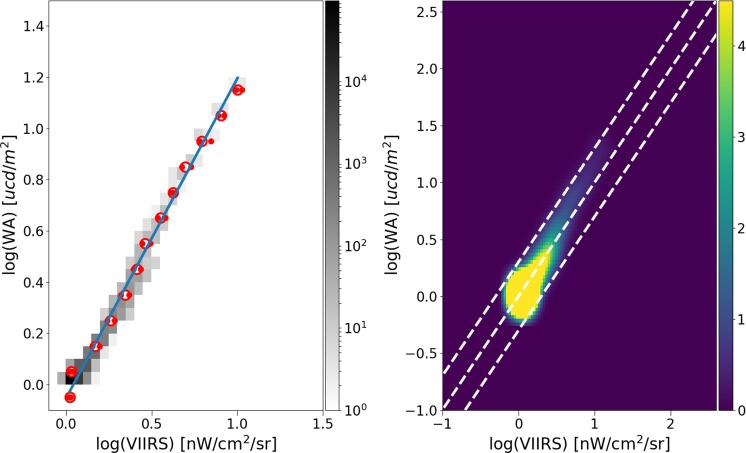
Correlation between the World Sky Atlas model^33^ and the observed median values from VIIRS. On the left the density plot and median values plus best fit. On the right, line 1:1 and parallel lines with ±0.3.

We compared the VIIRS data to the predictions of the World Sky Atlas^33^ in a region over the sea near Valencia and the Balearic Islands. This area has no interference due to direct lights, and also has low albedo. To increase the signal to noise ratio of the VIIRS data, we used the median of the four months closest to the second equinox (Aug to Nov), and then averaged the values from 2012 to 2018. We found an excellent fit between the model predictions and the observed values (Fig. 8).

## Discussion

The diffuse light around cities in nighttime satellite imagery of the Earth has often been interpreted as instrumental error. Here we have shown that this is not the case. Rather, this light is mostly due to sky brightness propagated from urban areas. This is quite an exciting result, as it means that, in principle, maps of sky brightness could be produced from satellite data.

The current state of the art in understanding the spatial variation of skyglow are maps produced by sky brightness models. While these are undeniably useful, the models have limitations, and in many cases experimental data would be preferred (e.g. for daily time series). Furthermore, obtaining sky brightness maps based on ground-based data is an extraordinary challenge. For example, Bara has demonstrated^38^ that interpolating a skyglow map accurately requires data with grid spacing of about 1 km. This would be difficult to accomplish with ground based observations, and is therefore the kind of problem that space-based mapping is well suited for.

Before maps of skyglow can be generated using space-based data, a better understanding of the relationship between skyglow observed from space and from the ground must be achieved. The variation between observed and DMSP predicted sky brightness reported here can be compared to that previously published by Kyba *et al*.^19^. In Kyba *et al*., the standard deviation of the residuals was ~0.5 mag_SQM_/arcsec^2^, whereas here we observed 0.3 mag_SQM_/arcsec^2^. A major difference between the two works is that Kyba *et al*. was based on observations obtained through a citizen science project. The improved accuracy in this paper can be attributed to several factors. First, the UCM sky brightness survey was performed by a single group of researchers using a single protocol and in similar weather conditions. In contrast, the citizen science data may include data from more turbid atmospheres, and the overly bright outliers in the citizen science data suggest that it was often taken too close to light sources. Also, the data from the UCM survey have been very carefully filtered to avoid false values. This issue is more difficult to address in a citizen science project.

When we analyze the residuals of all the observed sky brightness values and the predictions based on the fit between the satellite data and the UCM survey (see Fig. 5), we see the width of the main component is similar for all of them. The main difference between the different residuals is the shape of the tail to greater brightness. In the case of the DMSP data, the tail is not very pronounced, which we attribute to the large spatial size of its Point Spread Function (PSF). In the case of the other satellites, it is clear that the distribution is more asymmetric. (Note that for the DMSP satellite radiometer, the composite images do not show native resolution, but are rather based on averaging and super resolution methods^23^).

**Figure 9:**
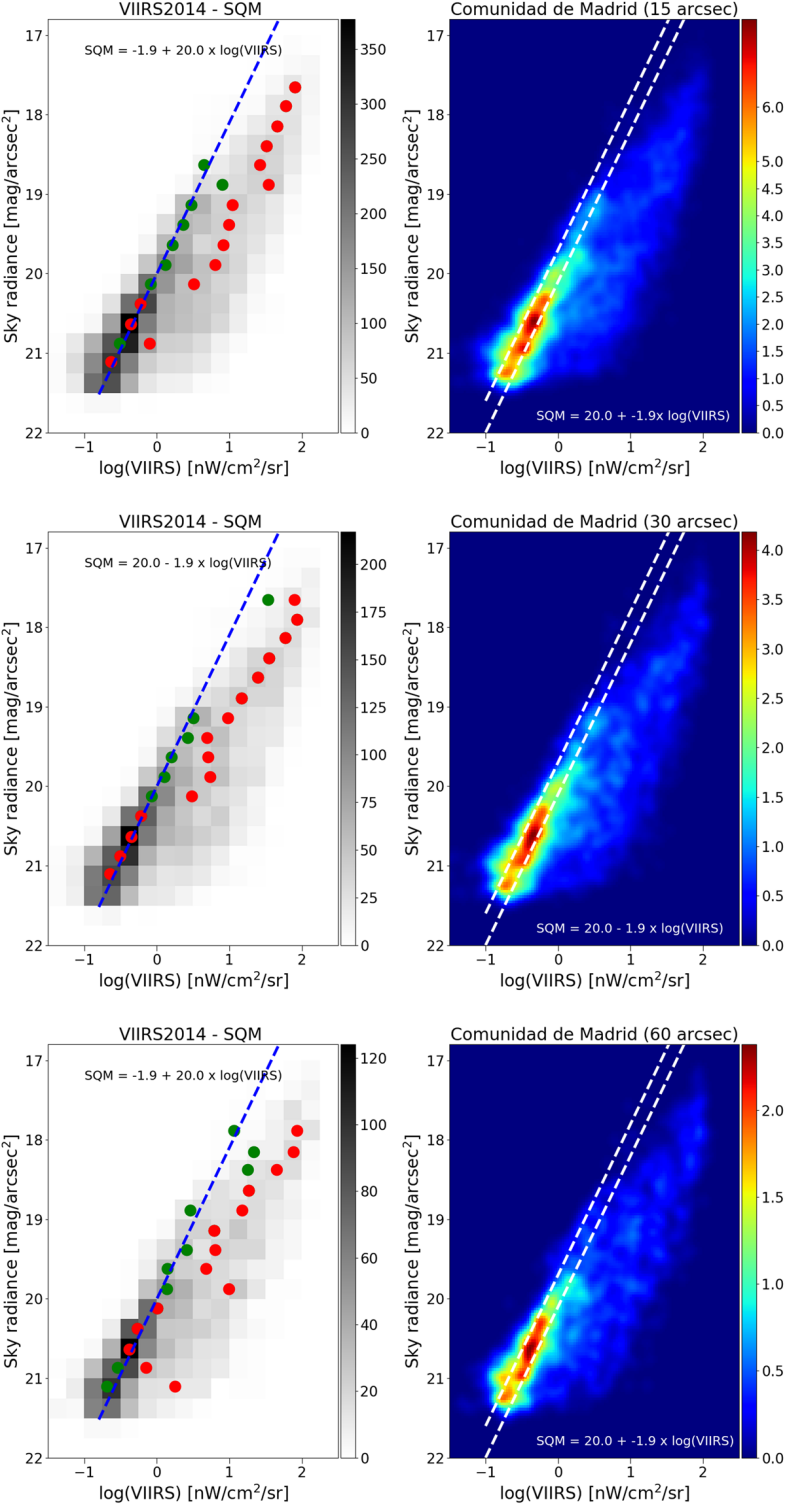
Relationship between sky brightness measured on the ground and satellite radiance observation for different sizes of pixel agglomeration for the VIIRS data. In the top plots, the VIIRS image data has been averaged over a pixel size of 15″ (arcseconds), in the middle plots 30″, and in the bottom plots 60″. In all cases, the relationship is strongest using the high resolution data. In the left plots, the green circle represents the dimmer Gaussian peak, and the red circles the brighter Gaussian peak. The right hand plots show the same result using a non-binned color coded density plot with a Gaussian filter. The two dashed lines have the same slope as the blue fit, and are shown for comparison purposes.

**Figure 10:**
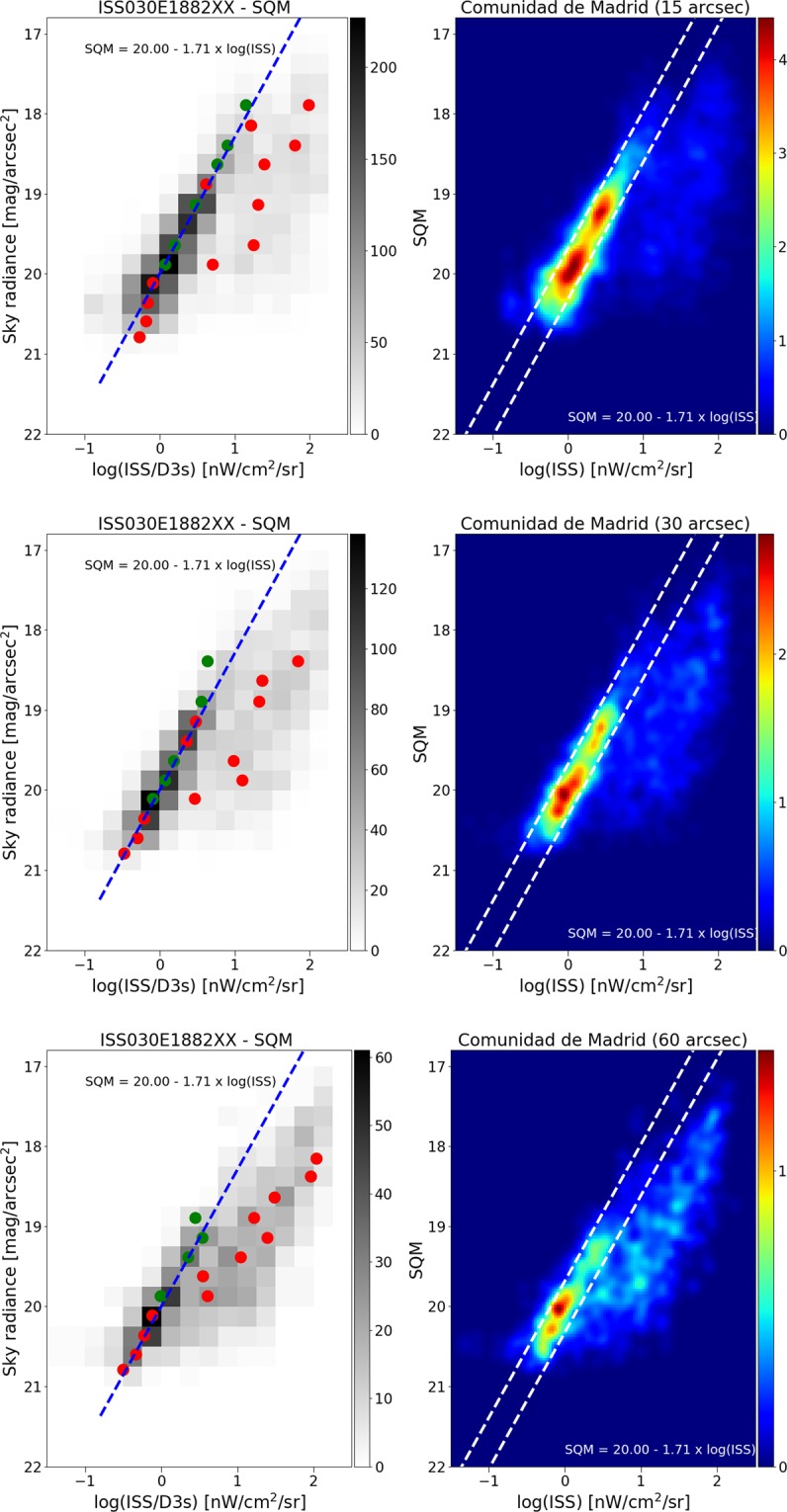
Relationship between sky brightness measured on the ground and satellite radiance observation for different sizes of pixel agglomeration, for ISS photo ISS030E1882. In the top plots, the ISS image data has been averaged over a pixel size of 15″ (arcseconds), in the middle plots 30″, and in the bottom plots 60″. In all cases, the relationship is strongest using the high resolution data. In the left plots, the green circle represents the dimmer Gaussian peak, and the red circles the brighter Gaussian peak. The right hand plots show the same result using a non-binned color coded density plot with a Gaussian filter. The two dashed lines have the same slope as the blue fit, and are shown for comparison purposes.

**Figure 11:**
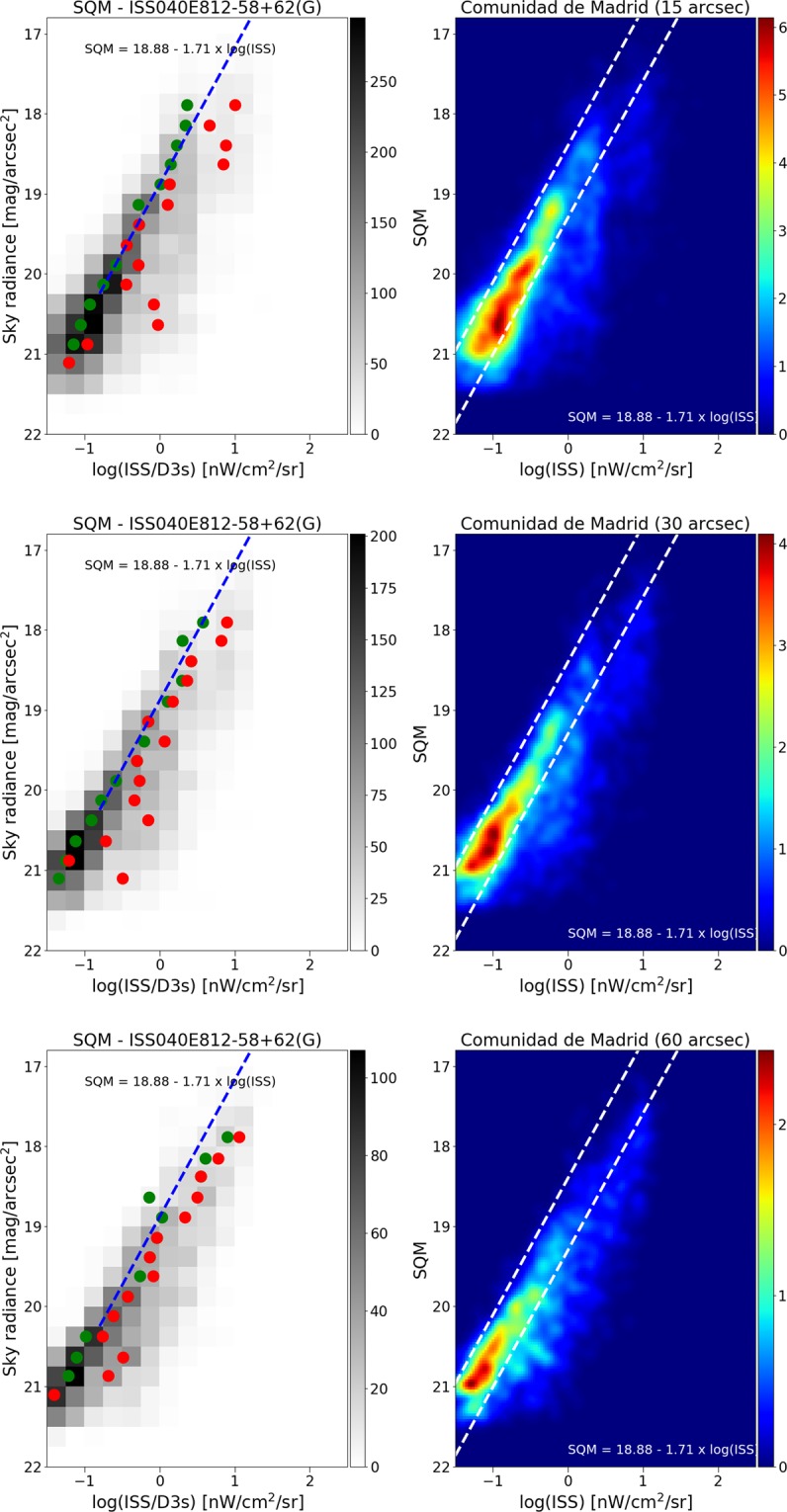
Relationship between sky brightness measured on the ground and satellite radiance observation for different sizes of pixel agglomeration, for ISS photo ISS040E0812. In the top plots, the ISS image data has been averaged over a pixel size of 15″ (arcseconds), in the middle plots 30″, and in the bottom plots 60″. In all cases, the relationship is strongest using the high resolution data. In the left plots, the green circle represents the dimmer Gaussian peak, and the red circles the brighter Gaussian peak. The right hand plots show the same result using a non-binned color coded density plot with a Gaussian filter. The two dashed lines have the same slope as the blue fit, and are shown for comparison purposes.

**Figure 12:**
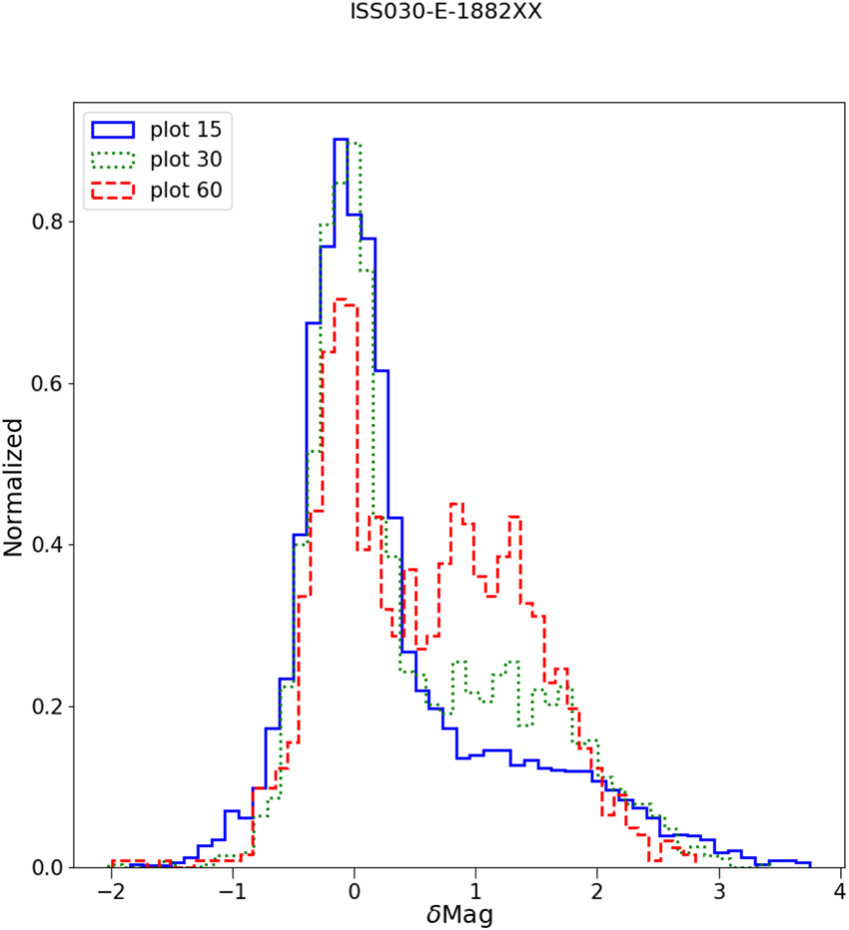
Impact of pixel agglomeration area on relationship between sky brightness and radiance detected from space. The histograms show the difference between the observed sky brightness and that predicted based on the ISS photograph. Larger values mean the sky is darker than would be expected. The relationship is best when the pixel size is smallest, or in other words, when the agglomerated pixel is small enough that it is unlikely to contain an admixture of both scattered skyglow and direct emissions.

We further examined what happens at lower spatial resolution, by producing reduced resolution maps from the different satellite datasets, and then comparing these to the SQM observations. In all cases, we resampled the satellite maps to 15 arcseconds, 30″, and 60″. The changing relationship to the SQM observations is shown for DNB in Fig. 9, and for the two ISS photos in Figs. 10 and 11. As the resolution is reduced (i.e. pixel size is increased), the tail begins to grow and the size of the main peak decreases. We attribute this effect to the admixture of direct illuminated and diffuse illuminated signal within the same pixel. The sky brightness is more strongly related to diffuse light than to direct illumination, so as resolution is reduced information is lost. Histograms of the fit residuals at different resampled resolutions are shown for one of the ISS images in Fig. 12.

We conclude that in the surroundings of Madrid and nearby municipalities, in most cases radiance values between 0.2–5 nW/cm^2^/sr are generally due to diffuse light. However, it is not possible to apply this as a land use classification, because direct light from small communities is frequently also in that range. Assuming that the Madrid data are representative of sky brightness in the rest of the world, this approach could be extrapolated more widely. As long as only broadband sensors are available, the correspondence between satellite radiance and skyglow will need to be adjusted locally, because the spectra of cities and types of light sources can differ.

**Figure 13:**
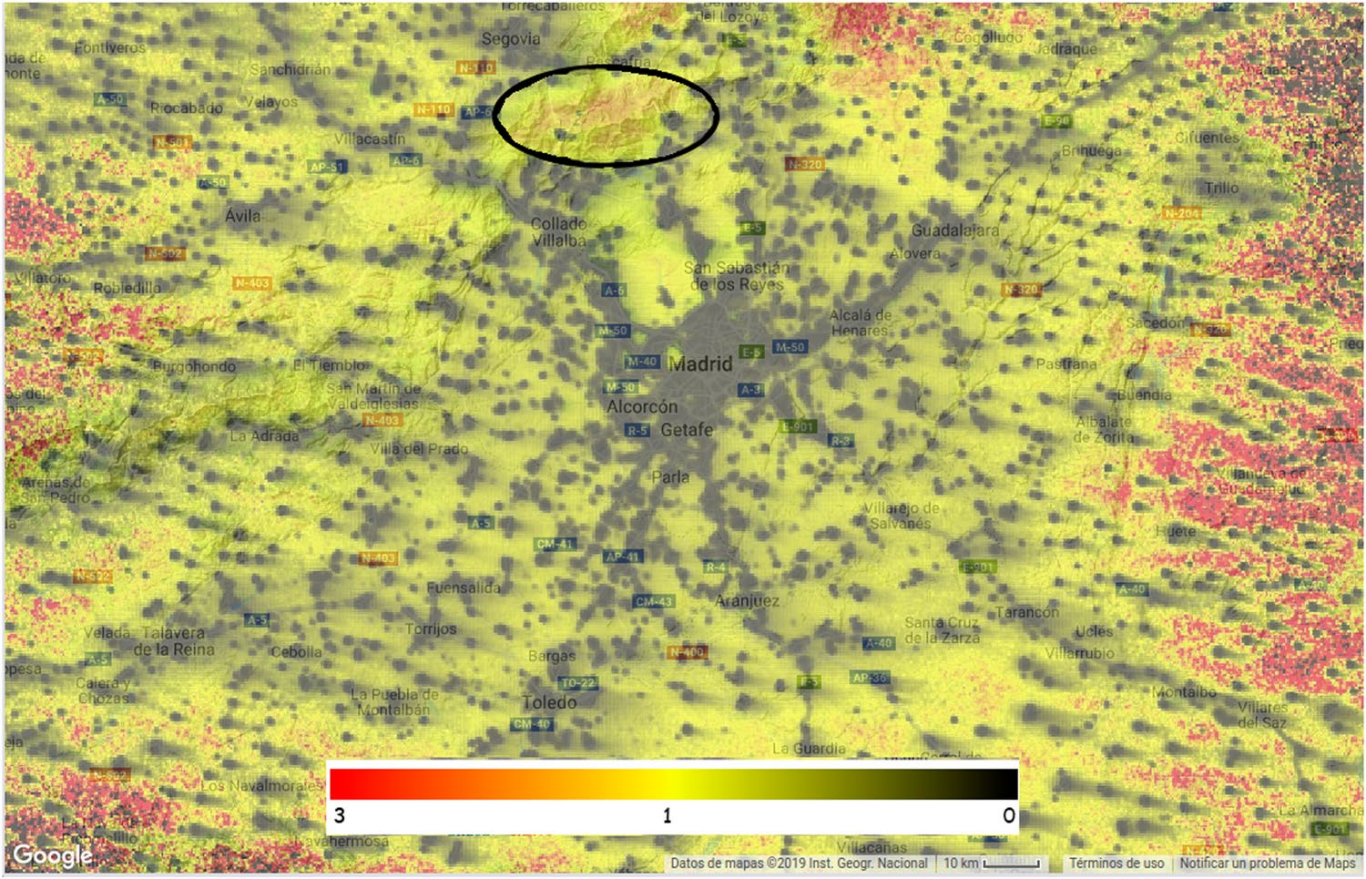
Ratio of our model of sky brightness based on VIIRS to the predictions of the World Atlas. In areas where VIIRS observes direct light the ratio is close to zero, as VIIRS is detecting direct rather than scattered light. In most areas near Madrid but outside of cities, the ratio is close to one. These are the areas where we believe VIIRS is observing skyglow, and values therefore match the World Atlas skyglow model. Some areas have higher ratios than one, for example the area North of Madrid which is circled. Our interpretation is that the World Atlas model is not correctly accounting for the blocking effect of mountains, as a large part of that area corresponds to the Sierra de Guadarrama National Park, and the Lozoya River basin, which is separated from Madrid by 1500 m high mountains. Computed with Google Earth Engine: https://pmisson.users.earthengine.app/view/tendsdiff (Source code available by request).

In order to remotely sense skyglow using satellite imagery, other aspects of nighttime imagery such as natural sky glow (auroras and airglow), reflectance of the ground, thin cloud and fog, and transient lights, will also need to be studied in more depth. New sky brightness surveys are needed in areas sensitive to effects not considered by^33^, such as blocking effects of orography. For example, the circled area in Fig. 13 shows a significant difference between our model of skyglow based on VIIRS and ISS data (Fig. 7) and the sky brightness reported by the World Atlas. We believe this is most likely due to the mountains blocking the light of Madrid, resulting in a darker sky beyond them.

Recently, Simons *et al*.^39^ compared sky brightness measured via all-sky imagery to the World Atlas as well as to VIIRS radiance data. They found a higher correlation to the World Atlas than to the satellite data. This is to be expected based on the data in Fig. 13, as pixels containing a mix of direct and scattered light do not have a simple correlation with sky brightness (Differences between the World Atlas and our predictions based on DNB data can be viewed at https://pmisson.users.earthengine.app/view/tendsdiff). It would be interesting to see a similar study to Simons *et al*., but in places which have no direct light emissions. Observations from water bodies such as done by Jechow *et al*.^40^ are promising, but we caution that such studies should be done at least 1 km away from shore lights.

### Temporal resolution

The focus of this paper is on the spatial distribution of skyglow, and not on temporal variation. However, light emissions are dynamic^41,42^, and produce changes in skyglow that can easily be detected^26,43–46^. This well known temporal variation acts as noise in this analysis, increasing the dispersion of the values. Sky brightness also changes over time due to changes in atmospheric properties (indeed, the relationship between diffuse light and the amount of aerosols^47^ provides additional evidence that the glow observed around cities is from real light).

Despite this, temporal variation can be effectively ignored here. Sánchez de Miguel^26^ presented an intensive analysis of the spatial, spectral and temporal variation of light pollution in Madrid and surrounding areas. The analysis found temporal variations with an amplitude of up to 0.3 magnitudes (~30%). Such variations are of second order compared with the spatial variation that can be up to 3 magnitudes (~factor 15).

The VIIRS, DMSP and ISS images also vary in terms of timing. The VIIRS data is based on the average statistics taken during several months, the DMSP data is based on the average statistics collected during a year, and the ISS images are nearly instantaneous samples at two moments in time. Ground based observations of skyglow can be either focused on temporal variation (stationary detectors) or spatial variation (with a moving detector). This study used moving detectors to cover a large area, and therefore the data were taken at different times in the night. Nevertheless, as discussed above, this effect is small compared to the spatial effect that is the key focus of this study.

Finally, we note that the DMSP data is taken during the early part of the night, whereas VIIRS data is taken well after midnight. If one were to restrict the comparison to skyglow data taken only during overpass times, it would greatly limit the spatial coverage that could be considered.

### Other components of the diffuse light: albedo, natural airglow, sea fogs and real blooming

It is beyond the scope of this article to address all of the components of dim diffuse light that may be present in VIIRS images. However, we wish to make clear that not all of the diffuse light can be explained by the scattering of artificial light. Brighter diffuse areas appear in VIIRS images for a number of reasons, including natural airglow and aurora, areas with higher ground albedo or fog, and even an instrumental effect that appears similar to CCD blooming.

**Figure 14:**
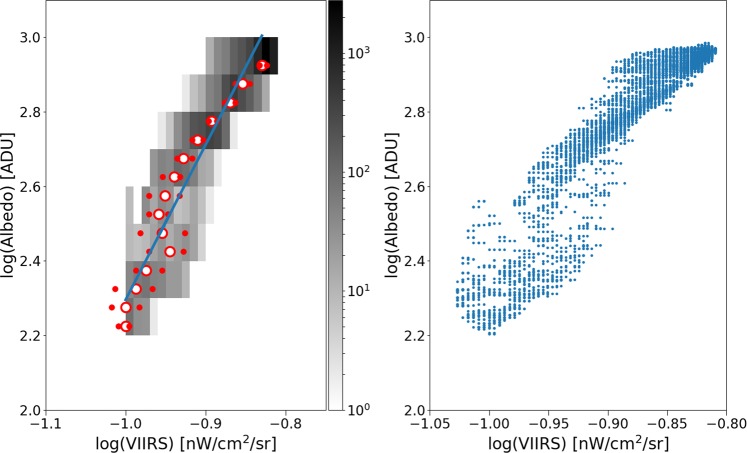
Correlation between the MODIS Albedo BSA Bands 1,2,3 and 4 and VIIRS median 2012–2018 in the proximity of the Wal al Namus volcano (Lybia).

The sensitivity of the VIIRS DNB is so high that it can detect the reflectance of starry skies on the ground. We show the impact of albedo in Fig. 14. The region near the Wal al Namus volcano experiences among the most extremely rapid changes of albedo on the surface of the Earth. The figure shows the correlation between the median of the Albedo bands of MODIS and the VIIRS DNB for the four months closest to the second equinox (Aug to Nov), with data from 2012 to 2018. A clear correlation was observed (*R*^2^ = 0.93), although there is some structure that indicates that the spectral sensitivities of the VIIRS and MODIS do not match perfectly, so this result could be further improved. This effect was previously reported by Roman *et al*.^48^.

The atmosphere naturally emits light that can be detected from the ground, primarily in a number of spectral lines^49^. These also contribute to sky brightness observed from space, but in a highly variable fashion because airglow is not constant from night to night. The area near the poles also has a permanent auroral ring around it.

Finally, higher than normal radiance is often observed in some areas of the ocean, and we believe this may be due to low level fog, thin clouds, or broken clouds that are smaller than the pixel size. These can be challenging to identify with the infrared channels of VIIRS, because they may have nearly the same temperature as the sea itself. The sea has a reflectance of less than 10%, while clouds have much higher albedos (e.g.^50^). There are often regions over the ocean with areas of diffuse light in the VIIRS monthly composites, and we believe this is due to fog or thin cloud that has not been identified in the cloud masking. This effect is most obvious near the equator between America and Africa, and in the northwest Pacific and northeast Atlantic (https://pmisson.users.earthengine.app/view/trends). This matches the areas of the Ocean that most often have cloud cover in the daytime VIIRS images (https://hannes.enjoys.it/carto/VIIRS_SNPP_CorrectedReflectance_TrueColor_median/).

**Figure 15:**
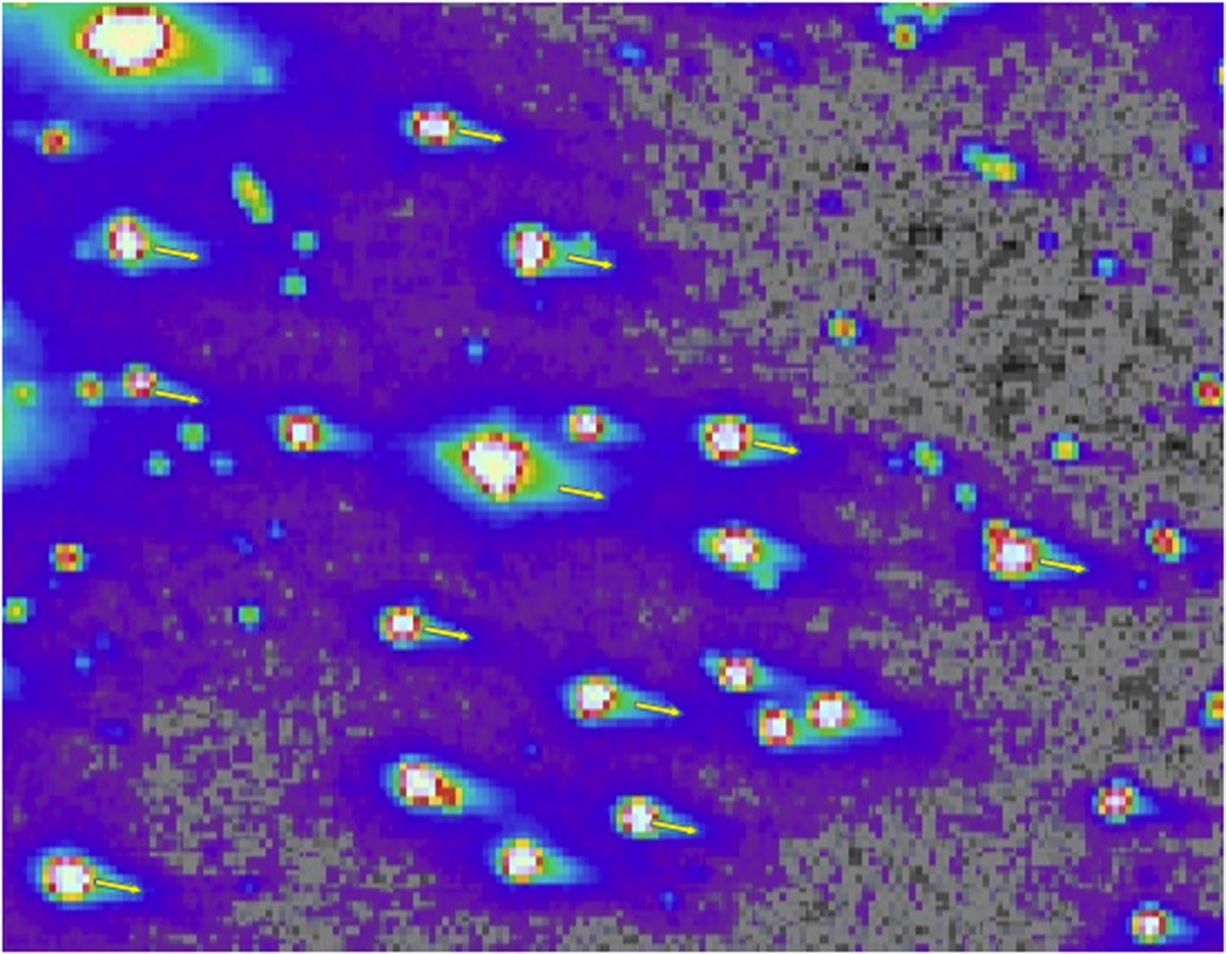
Blooming effect of dim sources near Villanueva de los Infantes, Spain (3.01°E, 38.65°N). This was based on finding the median value from 4 months in each calendar year, and then finding the mean of these values over 6 year.

Coming full circle, although the majority of diffuse light in nighttime imagery arises from sky brightening and not “blooming”, we have in fact occasionally observed an instrumental effect that appears similar to blooming. This is detectable in the high gain regime of VIIRS as tails to the distribution of brightness values that are a consequence of long exposure or blooming from the window of the instrument. This effect can be clearly seen in Fig. 15.

## Methods

### Ground based data

From 2009 to 2014, the UCM group undertook a sky brightness survey around the Madrid region with Sky Quality Meter photometers mounted onboard cars. The group collected over 30,000 individual measurements in 56 different expeditions encompassing different atmospheric conditions. This survey aimed to cover as much area as possible, while always having at least two sets of measurements per cell in different periods of the night and in different years whenever possible. In cases where it was not possible, the group aimed to at least have multiple measurements on the same transect in any given expedition. Precise time stamps are available in the dataset. Measurements were not taken during extreme conditions that could potentially interfere with sky brightness analyses, such as raised aerosol events like Saharan dust advection events, moonlight or clouds. The full dataset is openly available^51^. When the Region of Madrid is divided into cells of 2.2 km^2^, 60% of the total area is covered^18,26,52^. During this period there was no significant change in the overall street lighting power consumption in Madrid^53^.

### Space based data

In order to test the relationship between the diffuse light seen from the satellite and skyglow detected from the ground, we used all of the available data. The data from the SNPP/VIIRS/DNB and the DMSP/OLS are publicly available and produced by the Earth Observation Group (EOG). The skyglow and DMSP of the Madrid area were discussed in depth in Zamorano *et al*.^54^. The DMSP-OLS has a resolution of 5x5 km/pixel although the sampling is at ~1 × 1 km. The image used in this analysis was the F16_20100111-20110731_rad_v4 file from the Earth Observation Group of the National Oceanic and Atmospheric Administration website, corresponding to the average of the data over a year and a half (see Hsu *et al*. for details^55^). The data based on astronaut photographs from the ISS have been produced by us specifically for this analysis. The procedure has previously been described in detail by Sanchez^26^, and here we provide a brief review of the key information.

**Figure 16:**
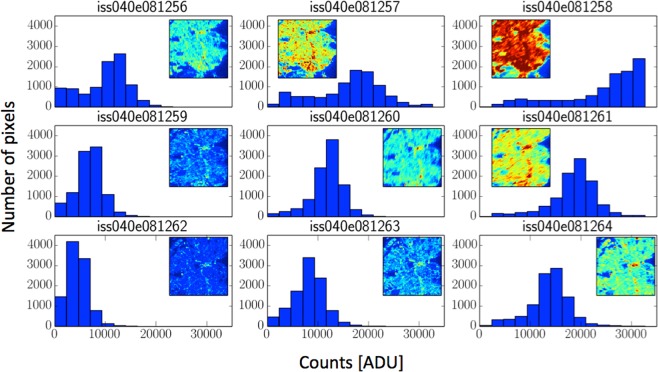
A series of histograms for images of Madrid taken using different camera settings by astronauts aboard the International Space Station during a single overpass. The images themselves are shown inset.

Like all sensors, the Nikon D3s cameras used on the ISS have a limited dynamic range over which the response to light is linear. This range depends on the camera settings (ISO, exposure time, aperture), and on the technology used in the sensor. In the case of the D3s, the linear range is between 100 to 10,000 “adimensional units” (ADU) in the raw file output (see details in Sanchez^26^). The relation between ADU and broadband spectral radiances depends on the settings used in the acquisition. One method to increase the dynamic range of acquisition is to combine different images of the same region taken with different settings. This technique is popularly known as High Dynamic Range Imaging (HDR). In our case, we made a selection of the known images of Madrid^24^, choosing those that provided the most information (Fig. 16). These images are photographs of Madrid on the same ISS overpass with different settings. The histograms show the distribution of ADU in the raw images, and it can be seen that in image ISS040-E-081258 the city is overexposed but the outlying regions are in the linear region, while in image ISS040-E-081262 the outlying regions are underexposed but most of the city lights are in the linear region. This pair of images also represent the extreme values of the time of exposure of the set, where all the rest of the settings are the same for the rest of the images.

This technique was used on two pairs of ISS images: ISS030-E-188208 and ISS030-E-188210 from March 28, 2012, and ISS040-E-081262 and ISS040-E-081258 from July 26, 2014 (the sky brightness data are compared to each HDR image separately). The long exposure time of 125 milliseconds (low intensity detected) used for the set ISS040-E-081258 image was only possible because the acquisition was made with the help of the Nightpod^56^. This device compensates for the movement of the ISS over the Earth, allowing longer exposure times without blurring the images. The images were acquired using the bracketing technique of taking sequentially several images with different time exposures. This technique was suggested as useful to create HDR images by^57^.

The UCM sky brightness survey was used for the calibration of the “World Atlas of artificial sky brightness”; Figures 17 and 18 and table 3 of Falchi *et al*.^33^ demonstrate that the sky brightness of Madrid is similar to that observed in other areas of the world for which observations are available. The impact of the different spectral responses of the different sensors (SNPP/VIIRS/DNB, DMSP/OLS, ISS and SQM) has been discussed by Sánchez de Miguel *et al*.^26,29^. Although this could produce differences in the observations between the instruments, for stable emission spectra from lights on the ground this could not result in differences larger than 0.5 mag for non-thermal emissions. As the typical streetlights in the region of interest did not change significantly during the 5 years during which the sampling campaigns were undertaken, the different spectral responses cannot explain the tail seen in Fig. 5. We attribute it instead to the presence of direct lights. Although the spectrum of sky brightness depends to some extent on wavelength because of Rayleigh and Mie scattering, the sensors all make broadband observations, and the main light source in the images is sodium lamps, for which the emission spectrum is dominated by a strong emission line near 590 nm.

### Fitting

Two different strategies were used to fit for a relationship between the satellite and ground based dataset, depending on which satellite dataset was considered. This is because of the different shapes for the fit residuals for the different satellite datasets (Fig. 5). For the low-resolution DMSP, the fit residuals have a single, clear peak. While this peak is not normally distributed, the unimodal shape means that the median value is stable. We binned the sky brightness data into bins of 0.5 mag_SQM_/arcsec^2^, and examined the histogram of DMSP radiances. The median radiance was assigned to the bin, and we performed an ordinary least squares fit on the data in the range 18.5–20.5 mag_SQM_/arcsec^2^.

The fitting procedure was slightly different for the higher resolution datasets. In this study, ground based observations of the sky always consist entirely of diffuse scattered light. Observations from space, however, sometimes contain only diffuse light (when the pixel has no light sources) but sometimes contains a mix of diffuse and direct light (when the pixel has some light sources). We wished to perform a fit that considered only the cases where the satellite viewed diffuse light. To do this, we performed a RANSAC linear fit^58^. RANSAC identifies outliers while performing the fit, effectively using only the data from the main peaks (e.g. Fig. 6) to perform the fit.

At the request of a reviewer, we examined the *R*^2^ of the full dataset (with outliers removed). We defined any observation that was more than 0.8 mag_SQM_/arcsec^2^ brighter than the prediction to be an outlier (this occurs because of the mix of direct and scattered light). We found that the *R*^2^ depends on the resolution of the satellite data, ranging typically from 0.75 to 0.85. The *R*^2^ was lowest in the cases when we reduced the satellite data to 60″ resolution, and highest at 15″ resolution.

In order to verify that the RANSAC fit was successful, we compared it to an alternate technique. We binned the ground based data in 0.5 mag_SQM_/arcsec^2^ bins, in order to acquire histograms of satellite-measured radiances. We then fit these individual histograms with a two-Gaussian fit, similar to that in Fig. 5. The Gaussian centered at lower radiance is made up of pixels which only contain diffuse light, whereas the broader Gaussian at higher radiance comes from pixels that include both diffuse and direct light.

The results of these fits are indicated by blue and red circles in Figs. 9–11. In cases where the fit returned two Gaussians, the fit centroid of the Gaussian at lower radiance is shown with a blue circle, and the higher radiance is shown with a red circle. In cases where the fit returned only one Gaussian, only a red circle is shown. The fit using RANSAC is shown as a dashed blue line, and for all three space based datasets, the RANSAC fit agrees well with the centroid of the lower radiance Gaussian.
